# Accessory Breast Cancer Occurring Concurrently with Bilateral Primary Invasive Breast Carcinomas: A Report of Two Cases and Literature Review

**DOI:** 10.7497/j.issn.2095-3941.2012.03.008

**Published:** 2012-09

**Authors:** Jin-yan Hao, Cui-cui Yang, Fang-fang Liu, Yi-ling Yang, Shuai Li, Wei-dong Li, Ya-qing Li, Rong-gang Lang, Yu Fan, Estifanos Paulos, Xin-min Zhang, Li Fu

**Affiliations:** 1 Department of Breast Cancer Pathology and Research Laboratory, Tianjin Medical University Cancer Institute and Hospital, Key Laboratory of Breast Cancer Prevention and Therapy, Ministry of Education, Tianjin 300060, China; 2 Department of Pathology and Laboratory Medicine, Temple University Hospital, Philadelphia 19140, USA

**Keywords:** invasive breast cancer, bilateral, primary, accessory breast cancer, occurring concurrently

## Abstract

The development of accessory breast tissue, which is found anywhere along the milk line, is attributed to the failure of milk line remnants to regress during embryogenesis. Primary tumors may arise from any ectopic breast tissue. Accessory breast cancer occurring concurrently with primary invasive breast cancer is extremely rare. Two such cases were reported in this article. One was a 43-year-old Chinese female who exhibited bilateral breast cancer (invasive ductal carcinoma, not otherwise specified, IDC-NOS) and an accessory breast carcinoma (IDC-NOS) incidentally identified in her left axilla. The ectopic breast tissue in her right axilla presented with adenosis. The patient was surgically treated, followed by postoperative docetaxel epirubicin (TE) chemotherapy. The second case was a 53-year-old Chinese female with bilateral breast cancer (apocrine carcinoma) accompanied by an accessory breast carcinoma (IDC-NOS) in her right axilla that was also incidentally identified. The patient was surgically treated after three doses of cyclophosphamide epirubicin docetaxel (CET) neoadjuvant chemotherapy, followed by adjuvant chemotherapy of the same regimen.

## Introduction

Breast tissues develop from the ectodermal ridges, also known as the milk lines, on the ventral surface of the body, which extend from the axillae to the inguinal regions and end on the medial aspect of the thighs on both sides of the body^[^^1^^]^. They spontaneously regress during embryogenesis, except for the pair at the pectoral region that forms mammary tissues in adults. Literature reports on accessory breast cancer developing from ectopic breast tissues are not infrequent. However, the presence of an accessory breast cancer concurrent with breast cancer is extremely rare ^[^^2^^, ^^3^^]^. Moreover, the presence of accessory breast cancer with bilateral invasive breast cancer has not yet been reported. We describe two such cases in this article, to add valuable information about this rare tumor to the current literature.

## Case Reports

### Case one

A 43-year-old Chinese female visited a doctor in our hospital after noticing a mass in each side of her breasts for 2 weeks. No family history of breast cancer was noted. Clinical examination revealed a 4-cm tumor in the left breast. The tumor was immobile, solid, and irregular, with apparently enlarged left axillary lymph nodes forming a 2-cm firm and immobile mass with irregular boundary. A 3-cm immobile, solid, and irregular mass was also found in the right breast, with palpable right axillary lymph nodes forming a 1.3-cm soft mobile mass of regular boundary. The skin and nipple of the patient’s breasts were unremarkable. Mammography (MMG) and ultrasonography (US) evaluations confirmed the physical findings and reported the bilateral breast masses and the left axillary lesion suspicious for malignancy. Core needle biopsy yielded the diagnosis of invasive ductal carcinoma in both breasts. The patient underwent bilateral modified radical mastectomy, followed by postoperative docetaxel epirubicin (TE) chemotherapy.

**Figure 1:**
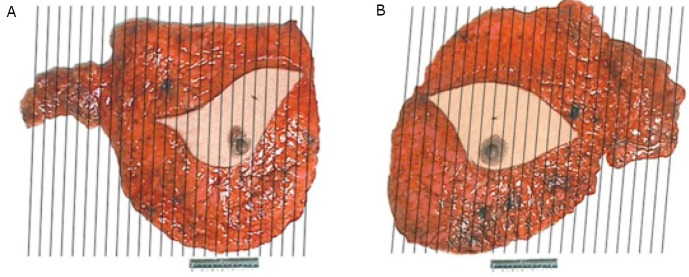
Case one. Mastectomy specimens and the dissection method: right (A) and left (B).

Mastectomy specimens with axillary contents were placed en bloc in formalin for 48 hours, and then dissected. The right mastectomy specimen measured 24 cm × 21 cm × 3 cm (**Figure 1A**). A mass measuring 1.6 cm × 1.5 cm × 1.2 cm was identified in the upper-inner quadrant, which was 4.5 cm away from the nipple. The cut surface of the tumor showed firm and dark red tissue with irregular boundary. Twelve lymph nodes ranging from 0.2 cm to 2.6 cm in greatest dimension were found in the right axilla. A soft tissue mass measuring 2.7 cm, which didn’t appear to be a lymph node, was also noted on the cut surface. The left mastectomy specimen measured 30 cm × 24 cm × 2 cm (**Figure 1B**). A 4.5 cm × 3 cm × 2.5 cm mass was located in the 12 o’clock position of the breast, 3 cm away from the nipple. The mass was firm with an irregular boundary on the cut surface. Fourteen lymph nodes ranging from 0.2 cm to 2.5 cm in greatest dimension were identified in the left axillary content. In addition, a 2.5-cm mass was found in the left axilla. This mass was far from the breast parenchyma and the texture of its tissue was different from that of the other lymph nodes on the cut surface.

**Figure 2:**
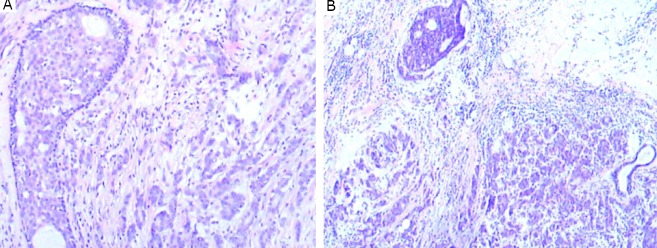
Ductal carcinoma in situ was admixed with the IDC-NOS in the right breast (A) (H&E stain, ×100). Ductal carcinoma in situ was adjacent to the IDC-NOS in the left breast (B) (H&E stain, ×40).

**Figure 3:**
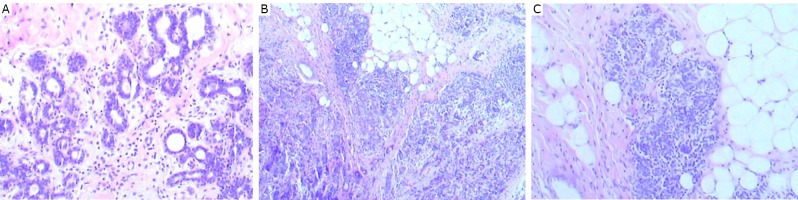
The right axillary accessory breast tissue exhibited adenosis (A) (H&E stain, ×100). IDC-NOS (lower left) and normal breast tissue (middle to upper) were identified in the left axillary accessory breast tissue (B) (H&E stain, ×40). The enlargement of the normal breast tissue in B is illustrated in C (H&E stain, ×100).

Microscopic examination revealed that the tumors in the right (**Figure 2A**) and left (**Figure 2B**) breasts were invasive ductal carcinoma, not otherwise specified (IDC-NOS), histologic grade II. Ductal carcinoma in situ was also noted to be admixed with and adjacent to the invasive carcinoma in each breast. The diagnosis was made according to the World Health Organization 2003 criteria^[^^4^^]^. The tumors spread to one of the 12 lymph nodes in the right axilla, whereas the 14 lymph nodes in the left axilla were spared from metastasis. The right axillary soft tissue mass was ectopic breast tissue with adenosis (**Figure 3A**). The left axillary mass was an IDC-NOS, histologic grade II, adjacent to benign ectopic breast tissue (**Figures 3B and C**). No lymphoid tissue was identified in the mass lesion. The mass was far from the pectoral breast parenchyma.

All invasive carcinomas were assessed by immunohistochemistry for estrogen receptor (ER) (SP1, Zymed), progesterone receptor (PR) (SP2, Zymed), human epidermal growth factor receptor 2 (HER2) (3B5+e2-4001, NeoMarkers), Ki67 (SP6, Zymed), and protein 53 (P53) (DO-7, Zymed) following standard procedures. The right breast tumor exhibited a positive reaction to ER (50%) and PR (15%), but was negative for the expression of P53 and HER2. This tumor presented a Ki67 labeling of 10%. The left breast tumor had a similar immunoprofile for ER (75%), PR (15%), and Ki67 (10%), but negative for P53 and HER2. The immunoprofile of the left axillary accessory carcinoma was different from those of the breast carcinomas, with a remarkably stronger positive reaction to PR (70%). However, the reaction of the left accessory carcinoma to ER, P53, HER2, and Ki67 labeling was similar. All tumors were classified as luminal A in the molecular subtyping.

### Case two

A 53-year-old Chinese female visited our hospital after noticing a mass in her left breast for one year and another mass in her right breast for five years. No family history of breast cancer was noted. Clinical examination showed a 4-cm left breast tumor, which was immobile, solid and irregular, without significantly enlarged accessible left axillary lymph nodes. A 4.5-cm immobile, solid, and cauliflower-like mass was found in the areola of the patient’s right breast. This mass exhibited skin ulceration and apparently had enlarged right axillary lymph nodes that formed an approximately 5-cm firm and immobile mass with irregular boundary. MMG and US results showed the bilateral breast masses and right axillary lesion suspicious for malignancy. Moreover, the left axillary lymph nodes were also reported to be suspiciously involved. Apocrine carcinoma was diagnosed in both breasts by core needle biopsy, and IDC-NOS was also found in the patient’s right axillary lesion. After 3 doses of cyclophosphamide epirubicin docetaxel (CET) neoadjuvant chemotherapy, the patient underwent bilateral modified radical mastectomy followed by adjuvant chemotherapy of the same regimen.

**Figure 4:**
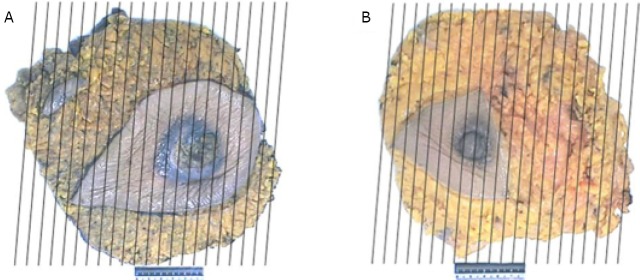
Case two. Breast mastectomy specimens and the dissection method: right (A) and left (B).

Mastectomy specimens with axillary contents were also placed en bloc in formalin for 48 h, and were then dissected. The right mastectomy specimen measured 20.5 cm × 19 cm × 4.5 cm (**Figure 4A**). A mass measuring 1.8 cm × 1.2 cm × 0.5 cm was identified in the areola of the breast. The cut surface of the tumor revealed firm and gray tissue with irregular boundary. Seventeen lymph nodes ranging from 0.3 cm to 2.5 cm in diameter were found in the right axilla. A 2.1-cm mass was also noted in the axilla. This mass was far from the breast parenchyma and its tissue texture was different from that of the other lymph nodes on the cut surface. The left mastectomy specimen measured 22 cm × 18 cm × 2 cm (**Figure 4B**). A mass measuring 1.5 cm × 1 cm × 0.7 cm was located on the side of the nipple. The mass was firm, with irregular boundary on the cut surface. Sixteen lymph nodes ranging from 0.3 cm to 1.8 cm in diameter were identified in the left axillary content.

**Figure 5:**
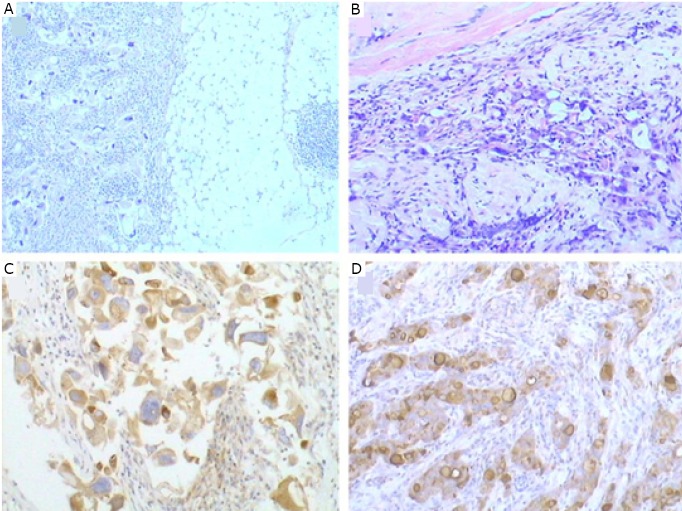
The tumors in the right (A) (H&E stain, ×40) and left (B) (H&E stain, ×100) breasts were apocrine carcinoma and chemotherapy reaction II. GCDFP-15 protein was positive in the right (C) and left (D) tumors (IHC LSAB, ×100).

**Figure 6:**
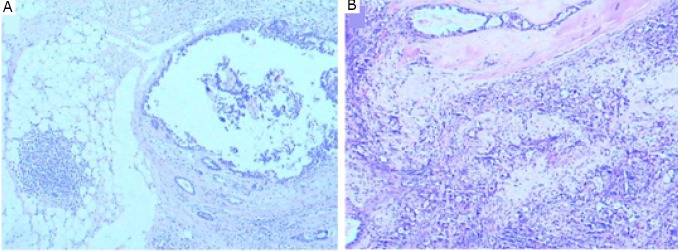
Ductal carcinoma in situ was adjacent to the apocrine carcinoma of the right breast (A) (H&E stain, ×40). Ductal carcinoma in situ was admixed with the apocrine carcinoma of the left breast (B) (H&E stain, ×40).

**Figure 7:**
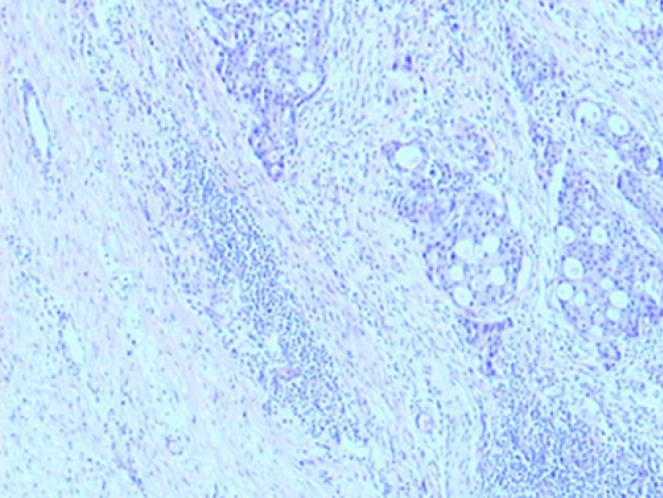
The right axillary mass consisted of IDC-NOS (upper right) with chemotherapy reaction II, adjacent to benign ectopic breast tissue (upper left), (H&E stain, ×40).

Microscopic examination revealed that the tumors in the right (**Figure 5A**) and left (**Figure 5B**) breasts were apocrine carcinoma, with GCDFP-15 protein expression in each tumor (**Figures 5C and D**). These carcinomas exhibited grade II chemotherapy reaction in the Miller-Payne system^[^^5^^]^. Ductal carcinoma in situ was also noted admixed with and adjacent to the invasive carcinoma in each breast (**Figures 6A and B**). The tumors spread to 1 of the 17 lymph nodes in the right axilla, and to 6 of the 16 lymph nodes in the left axilla. The right axillary mass consisted of IDC-NOS, with grade II chemotherapy reaction, adjacent to benign ectopic breast tissue (**Figure 7**). No lymphoid tissue was identified in the mass lesion. The mass was far from the pectoral breast parenchyma.

The invasive components of carcinoma were also assessed for hormonal and proliferative marker expressions following the same immunohistochemistry procedures used in case one. The right breast tumor was negative for ER (<1%) and PR (<1%), and exhibited a HER2 reaction with an intensity of 2+. The tumor was positive for P53, with 90% of nuclei stained and the Ki67 labeling index was 15%. The right axillary accessory breast carcinoma was negative for ER (<1%) and PR (<1%), and showed stronger HER2 reaction (3+). This carcinoma had a lower P53 labeling (70%) and a higher Ki67 labeling (50%). The left breast tumor was negative for ER (<1%) and PR (<1%), and positive for HER2 (3+), with a Ki67 labeling index of 20% and a P53 labeling index of 70%. HER2 gene amplification was identified in all tumors via fluorescence in situ hybridization (FISH). All tumors were classified as HER2+ in molecular subtyping.

## Discussion

The mammary gland develops from the ectodermal layer during embryogenesis. An ectodermal ridge, also called milk line, extending from the axilla to the groin area, appears on the sixth week of gestation^[^^6^^]^. The milk line normally disappears, except in the thoracic region, where the normal breast tissues will develop. However, breast tissues will develop in the ectopic area if the milk line remnants fail to regress during embryogenesis.

The presence of accessory breast tissue is uncommon, with an overall incidence range of 0.22% to 6.0% for the general population^[^^7^^,^^8^^]^. Women are reported to exhibit a higher rate than men. The most common location of ectopic breast is the axilla. Other less common locations are the face, thighs, perineum, groin, vulva, and shoulders^[^^6^^,^^9^^-^^11^^]^. Ectopic breast may consist of breast parenchyma, areola, and nipple, or any combination of these three components. This type of breast is subject to hormonal influences and undergoes physiologic changes. In addition, various pathological changes, including invasive carcinoma, may develop in these ectopic breast tissues^[^^12^^-^^15^^]^.

An accurate diagnosis of axillary accessory breast carcinoma is important because it can provide precise staging information for patients with concurrent ipsilateral breast cancer. However, this diagnosis can be extremely difficult, if not impossible, because primary breast cancer frequently extends to the axillary tail and metastatic carcinoma can completely replace involved lymph nodes. Therefore, axillary breast tail cancer, axillary lymph node tumor metastasis, occult breast cancer, and lymphoma have to be excluded before the diagnosis can be firmly established^[^^16^^]^. Differences in morphological features and in ancillary studies can provide helpful evidence in making the diagnosis. In the 2 cases discussed above, the fact that the accessory breast tissue was found in the axillae, grossly far from the pectoral breast parenchyma, favored the diagnosis of accessory breast carcinoma. More importantly, the ductal carcinoma in situ admixed with and adjacent to invasive carcinomas in the breasts established the diagnosis of primary bilateral breast cancer. The identification of normal breast tissue adjacent to the invasive carcinoma in the axilla strongly suggested invasive carcinoma arising in the ectopic breast tissue. The absence of accompanying lymphoid tissue further disfavored a diagnosis of tumor metastasis in the axillary lymph node from breast cancer. The accessory breast carcinoma in case one shared the same histologic type and expression profiles for ER, HER2, P53, and Ki67 with the primary breast carcinomas. However, a remarkably stronger PR expression seemed to distinguish the accessory breast carcinoma from the primary breast carcinomas. Meanwhile, the accessory breast carcinoma in case two shared molecular subtyping with the primary breast carcinomas, but with different histologic type, P53, and Ki67 expression. Notably, accessory breast cancer can be grossly similar to a lymph node with metastatic carcinoma. This fact should alert pathologists to pursue histologic examination of all tissue nodules in the axilla.

Similar with primary breast cancer, accessory breast cancer is also surgically treated and supplemented with preoperative or postoperative chemotherapy, radiotherapy, and endocrine therapy. Radical mastectomy or modified radical mastectomy is the preferred surgical procedure. Both patients in this study underwent bilateral modified radical mastectomies. The breasts, accessory breast tissue, and part of the adjoining pectoral major muscles were bilaterally removed. Radical axillary lymphadenectomy was carried out. Preoperative or postoperative chemotherapy is important in decreasing tumor size or eliminating residual tumors.
